# Impairing RAGE signaling promotes survival and limits disease pathogenesis following SARS-CoV-2 infection in mice

**DOI:** 10.1172/jci.insight.155896

**Published:** 2022-01-25

**Authors:** Forrest Jessop, Benjamin Schwarz, Dana Scott, Lydia M. Roberts, Eric Bohrnsen, John R. Hoidal, Catharine M. Bosio

**Affiliations:** 1 Immunity to Pulmonary Pathogens Section, Laboratory of Bacteriology, and; 2 Rocky Mountain Veterinary Branch, Rocky Mountain Laboratories, National Institute of Allergy and Infectious Diseases, NIH, Hamilton, Montana, USA.; 3 Division of Respiratory, Critical Care, and Occupational Pulmonary Medicine, Department of Internal Medicine, University of Utah, Salt Lake City, Utah, USA.

**Keywords:** Immunology, Infectious disease, Innate immunity

## Abstract

Cellular and molecular mechanisms driving morbidity following SARS-CoV-2 infection have not been well defined. The receptor for advanced glycation end products (RAGE) is a central mediator of tissue injury and contributes to SARS-CoV-2 disease pathogenesis. In this study, we temporally delineated key cell and molecular events leading to lung injury in mice following SARS-CoV-2 infection and assessed efficacy of therapeutically targeting RAGE to improve survival. Early following infection, SARS-CoV-2 replicated to high titers within the lungs and evaded triggering inflammation and cell death. However, a significant necrotic cell death event in CD45^–^ populations, corresponding with peak viral loads, was observed on day 2 after infection. Metabolic reprogramming and inflammation were initiated following this cell death event and corresponded with increased lung interstitial pneumonia, perivascular inflammation, and endothelial hyperplasia together with decreased oxygen saturation. Therapeutic treatment with the RAGE antagonist FPS-ZM1 improved survival in infected mice and limited inflammation and associated perivascular pathology. Together, these results provide critical characterization of disease pathogenesis in the mouse model and implicate a role for RAGE signaling as a therapeutic target to improve outcomes following SARS-CoV-2 infection.

## Graphical abstract

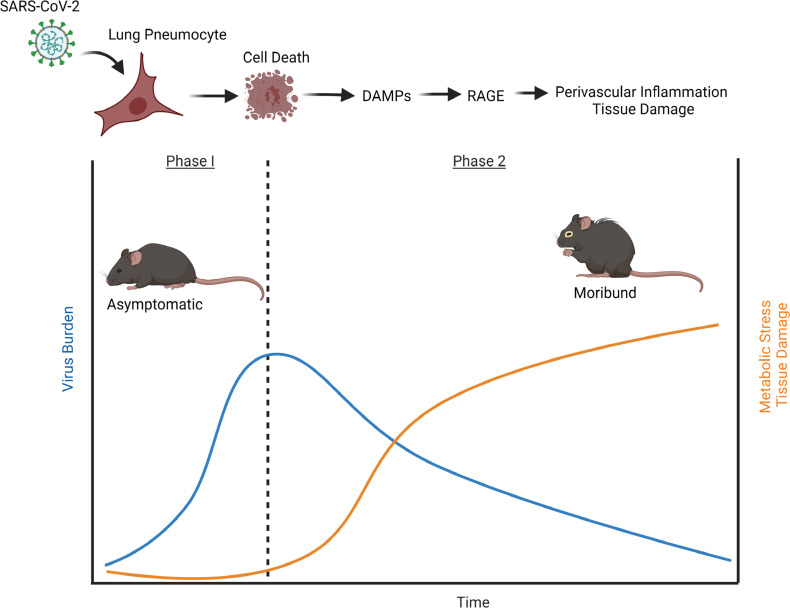

## Introduction

SARS-CoV-2 infection can lead to serious pulmonary and vascular tissue damage. Pathological reports indicate that diffuse alveolar damage, the presence of hyaline membranes, alveolar edema, and microthrombosis are hallmark features of advanced SARS-CoV-2 infection (1, 2). The etiology of lung injury following SARS-CoV-2 infection is not fully understood, but evidence has suggested involvement of hyperactive inflammatory and/or vascular coagulant responses (2, 3). Among patients admitted to intensive care units, mortality rates are reported to range between 26% and 61.5%. The mortality rate was further increased if patients were subjected to mechanical ventilation (4). There are limited therapeutic interventions available to treat SARS-CoV-2–associated lung injury beyond supportive care. Current treatment strategies for SARS-CoV-2 infection focused on targeting antiviral or antiinflammatory responses have shown conflicting or limited efficacy depending on when they are administered, in part because of our limited understanding of disease pathogenesis (5–7). An improved understanding of the pathogenesis of severe SARS-CoV-2 illness will inform optimal therapeutic intervention and reveal new molecular targets to treat disease.

One molecular target that has received significant attention as a possible therapeutic for SARS-CoV-2 infection is the receptor for advanced glycation end products (RAGE) (8–11). RAGE is a multiligand receptor that is highly expressed in the lungs on type I pneumocytes as well as several other lung cell populations including alveolar macrophages and endothelial cells (12, 13). Disruption of the alveolar/capillary barrier results in RAGE exposure to danger-associated molecular patterns (DAMPs) released from injured tissues, which activate proinflammatory and/or procoagulant signaling cascades (8, 14). RAGE signaling has also been reported to contribute to endothelial cell activation and vascular responses (15). Interest in targeting RAGE during SARS-CoV-2 infection has been further buoyed by strong evidence showing its central contribution to development of lung injury and associated vasculature dysregulation in several non–SARS-CoV-2 models (8, 16, 17). Furthermore, several DAMPs that activate RAGE signaling, including high-mobility group box 1 (HMGB1) and members of the S100 family, have been shown to be elevated during severe SARS-CoV-2 infection and correlate with poor prognosis (8, 18, 19). Prominent comorbidities associated with increased risk of serious SARS-CoV-2 infection, including hypertension and diabetes, also involve dysregulation of RAGE signaling (10, 11). However, the specific contribution of RAGE signaling to the development of lung injury following SARS-CoV-2 infection has not hitherto been investigated. In the current study, we impaired RAGE signaling in vivo and determined the contribution of this pathway to fatal SARS-CoV-2 infection.

## Results

### Characterization of SARS-CoV-2 disease pathogenesis in vivo.

**Figure 1:**
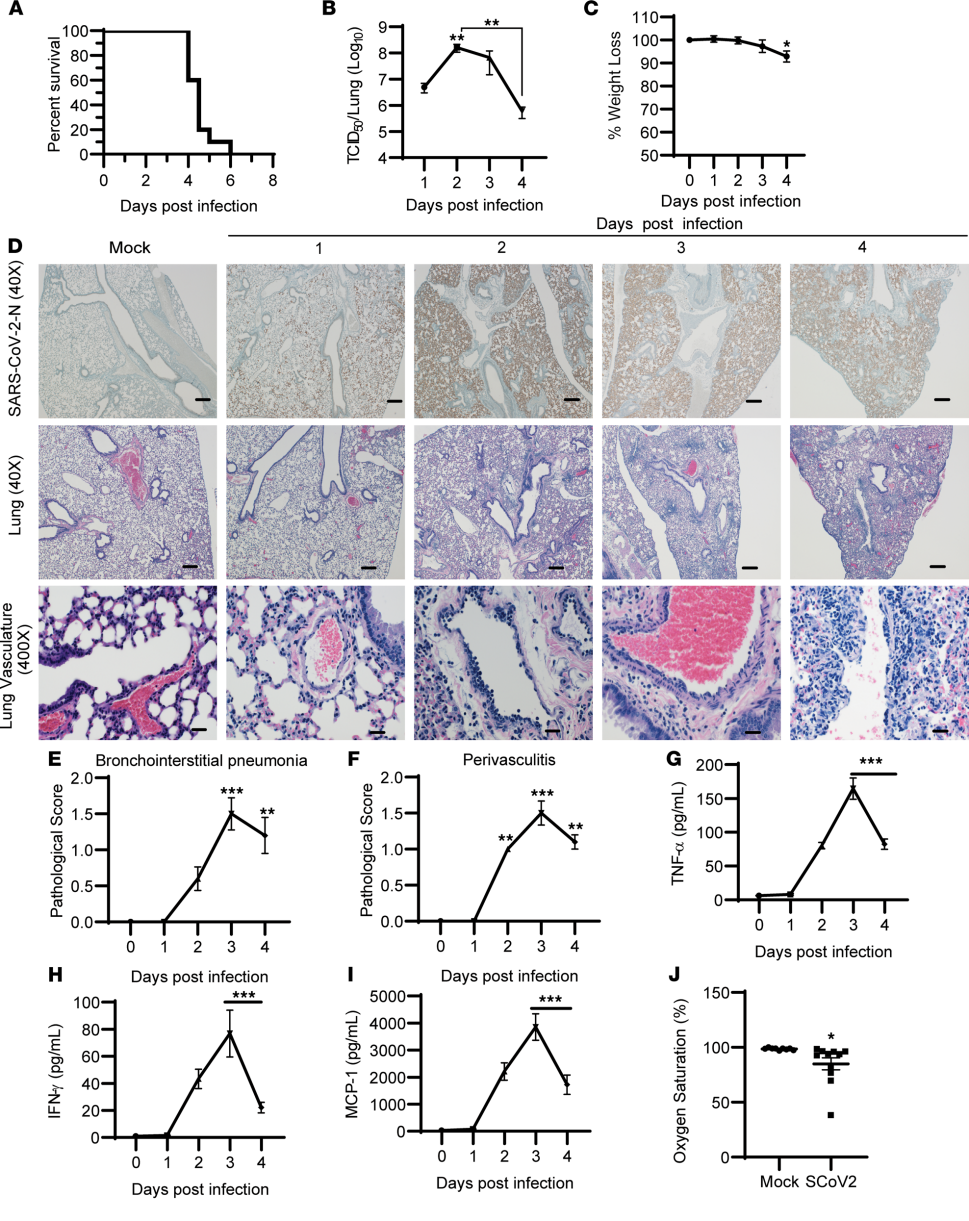
Characterization of SARS-CoV-2 disease pathogenesis in vivo. (**A**–**C**) Mice were infected by intranasal instillation with 1 × 10^4^ TCID_50_ SARS-CoV-2 and evaluated for survival (**A**), viral load by TCID_50_ (**B**), and weight loss (**C**). (**D**) Representative images of lung sections from SARS-CoV-2–infected mice stained for nucleocapsid antigen (×40) or H&E-stained for pathological analysis (×40 and ×400 magnification; scale bars: 20 μm at ×400 or 200 μm at ×40). (**E** and **F**) Lesions were scored between 0 (no lesion) and 5 (severe) for interstitial pneumonia (**E**) or perivascular cuffing (**F**). (**G**–**I**) Levels of the proinflammatory cytokines or chemokines TNF-α, IFN-γ, and MCP-1. (**J**) Oxygen saturation levels in mock- and SARS-CoV-2–infected mice on days 4–5 after infection. Survival data (**A**) are from *n* = 10 mice combined from 2 separate experiments. Otherwise, data are shown as mean ± SEM from *n* = 8 for mock-infected, *n* = 10 mice for infected groups, pooled from 2 separate experiments. *P < 0.05, **P < 0.01, ***P < 0.001 indicate significance compared with mock-infected (day 0) control using 1-way ANOVA or an unpaired, nonparametric Mann-Whitney test for histological scores.

K18-hACE2 mice have been successfully used to model both SARS and SARS-CoV-2 infection (20). However, reports vary with regard to lethal viral dose and mean time to death (21–24). Therefore, we first established and characterized the K18-hACE2 model in our laboratory, including lethality and mean time to death as well as the viral load and pathological response over time. K18-hACE2 mice were intranasally infected with 1 × 10^4^ TCID_50_ (50% tissue culture infective dose) SARS-CoV-2. Mice became moribund between days 4 and 6 after infection, with up to 80% of mice requiring humane euthanasia as early as 4.5 days after infection (Figure 1A). Evaluation of viral load over time in the lungs indicated that infectious virus peaked on day 2 after infection and declined thereafter (Figure 1B), consistent with prior reports (20). Weight loss in moribund mice was significant but marginal (mean = 7.1% ± 2.4%) (Figure 1C). Immunohistochemical analysis of SARS-CoV-2 N antigen paralleled viral replication at early stages of infection but remained elevated on days 3 and 4 after infection, despite a decline in infectious virus particles (Figure 1D). We next evaluated the pathological response in the lungs over the course of infection. Minimal to mild bronchointerstitial pneumonia was evident in infected mice on day 2 after infection (Figure 1, D and E). We did not observe significant alveolar edema or hyaline formation in moribund mice. Recent reports suggest vasculature involvement, including thrombosis, in the development of severe SARS-CoV-2 illness in both mice and humans (21, 25–27). Significant perivascular cuffing, appearing as early as day 2 after infection, was evident and involved endothelial cell hypertrophy (Figure 1, D and F). The perivascular cuffs were composed of small numbers of lymphocytes, with fewer macrophages and plasma cells, and rare neutrophils. Often, these inflammatory cells accumulated just under the endothelium of the affected vessel. Occasionally, similar inflammatory cell infiltrates extended into and thickened the alveolar interstitium (Figure 1D). Mice necropsied at 3 and 4 days after infection demonstrated very similar lesions; however, at the latter time point more mice in each group displayed interstitial pneumonia and perivascular cuffing and, in some cases, the severity and distribution of these lesions increased from minimal to mild (Figure 1, D–F). There was no evidence of thrombosis in the lungs of moribund mice. Evaluation of multiple proinflammatory cytokines showed cytokine production beginning to elevate on day 2 after infection, peaking on day 3 after infection, and decreasing by day 4 after infection, consistent with the pathological evaluation (Figure 1, G–I). Together these data indicate a dissociation between lung viral burden and inflammation with onset of morbidity.

Others have reported significant neurological involvement in K18-hACE2 mice potentially contributing to lethality (28). Thus, we also evaluated changes in brain tissue following infection. Unlike in previous reports, no significant tissue or vasculature pathology was observed in the brains of moribund K18-hACE2 mice by day 4 after infection (Supplemental Figure 1; supplemental material available online with this article; https://doi.org/10.1172/jci.insight.155896DS1). Examination of additional peripheral tissues revealed an absence of significant pathology in the spleen, kidney, or liver of infected mice relative to uninfected controls on day 4 after infection, suggesting that morbidity was not linked to pathological responses in these tissues (Supplemental Figure 1). However, moribund mice exhibited reduced oxygen saturation levels between days 4 and 5 after infection indicative of compromised pulmonary function despite the presence of only minimal to mild pathology and inflammation (Figure 1J). Therefore, it is possible that SARS-CoV-2 infection may be causing specific pathological responses affecting vascular function or dysregulation of other yet to be defined metabolic or cellular responses integral for efficient gas exchange.

### Metabolic stress indicative of inflammation and tissue damage occurs following peak viral burden.

**Figure 2:**
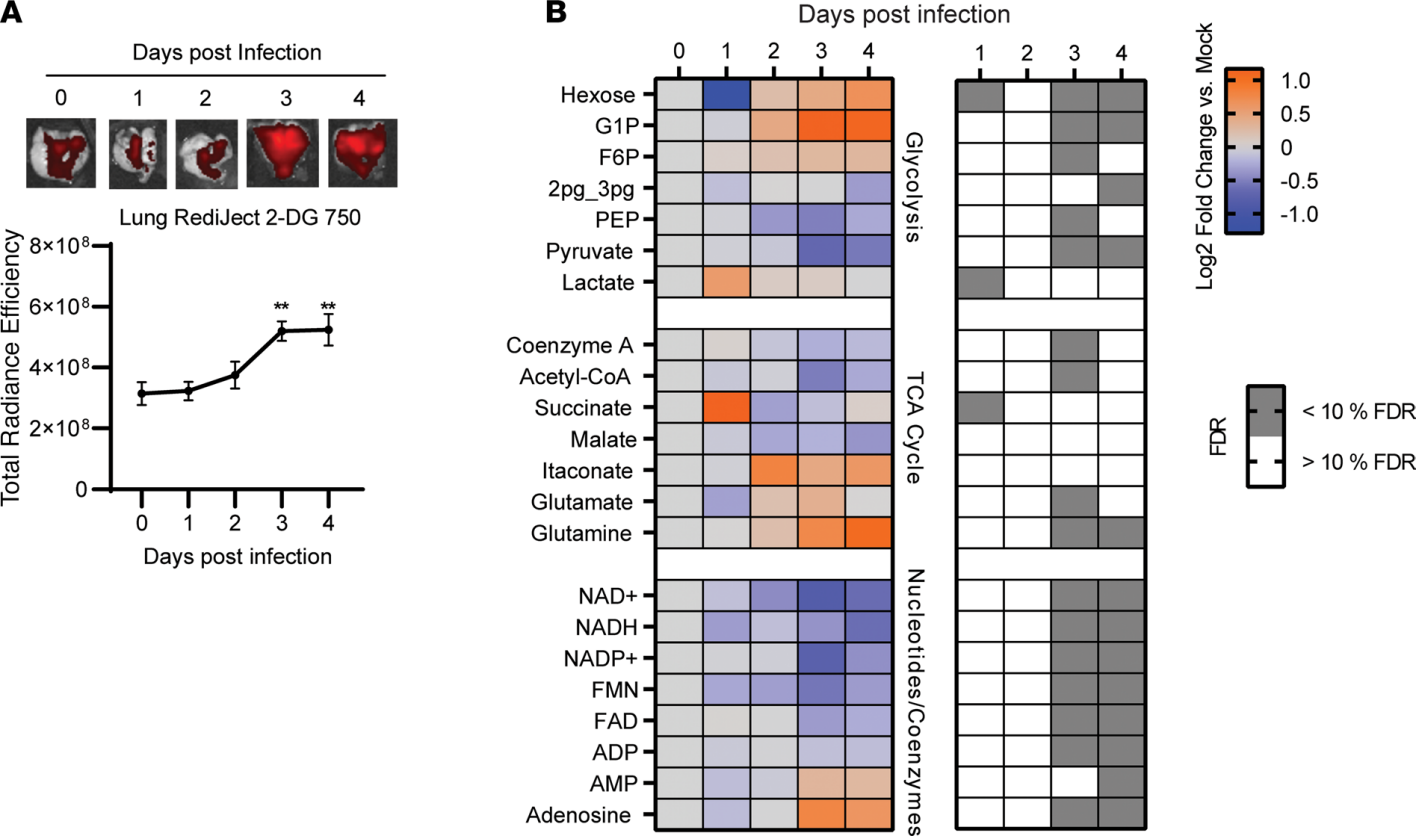
SARS-CoV-2 evades triggering metabolic reprogramming supportive of inflammation in vivo. (**A**) Ex vivo imaging of RediJect 2-DG uptake in the lungs of K18-hACE2 mice infected by intranasal instillation with SARS-CoV-2 (1 × 10^4^ TCID_50_). (**B**) Semitargeted analysis of soluble metabolites in whole lungs of SARS-CoV-2 associated with central metabolism including glycolysis and the TCA cycle. Data from experiments in **A** are shown as mean ± SEM (*n* = 9–10). Data in **B** are means of log_2_ fold change values relative to mock-infected (day 0) controls (*n* = 5 per group) and whether the corresponding false discovery rate was greater or less than 10%. **P < 0.01 indicates significance compared with mock-infected (day 0) control using 1-way ANOVA.

In humans, SARS-CoV-2 disease pathogenesis typically includes an asymptomatic phase associated with rapid viral replication, which can transition into a symptomatic phase involving progressive tissue damage (29). However, how and when tissue injury occurs in the lungs following SARS-CoV-2 infection, and what contribution that makes to morbidity, have not been well delineated. Tissue damage involves dysregulation and reprogramming of central metabolic processes including oxidative phosphorylation and glycolysis. Monitoring glycolysis can be an effective tool to temporally track disease pathogenesis and can reveal important molecular information regarding mechanisms of tissue injury (30). Accordingly, we measured the host glycolytic response in vivo as an additional predictor for tissue injury. We observed no upregulation of glycolysis, as assessed by accumulation of fluorescently labeled 2-deoxyglucosone (2-DG), during the phase of viral replication. However, a significant increase in glycolysis occurred in the lungs of infected mice on days 3 and 4 after infection relative to mock-infected (day 0) controls (Figure 2A), after peak viral burden was reached (Figure 1B). To further confirm whether metabolic reprogramming was occurring following peak viral burden, we assessed polar metabolites associated with central metabolism using mass spectrometry. Surprisingly, despite an absence of significant pathological changes and an absence of any major metabolic shift, isolated metabolic changes in glycolysis and TCA cycle intermediates, limited to glucose, lactate, and succinate levels, were evident as early as day 1 after infection (Figure 2B). However, broad perturbations in central metabolism were evident by day 3 after infection and were maintained through day 4 after infection, consistent with our 2-DG imaging results. Levels of the metabolite itaconate also were elevated by day 3 after infection, reflecting reprogramming of ETC complex II activity, increased host glycolysis, and inflammation (31). Additionally, we observed significant depletion in the NAD^+^ and NADH levels on day 3 after infection (Figure 2B). Maintaining NAD^+^ and NADH levels is critical for cell viability, and the loss of these cofactors can be indicative of tissue injury (32). Several other metabolic signatures of disease progression were also noted, including depletion of hypoxanthine and associated ribonucleotide inosine monophosphate and accumulation of most amino acids (Supplemental Figure 2). Together, the host metabolic response revealed dysregulation of central metabolic pathways that support inflammation and/or tissue damage.

### Temporal dynamics of death in lung cell populations following infection.

**Figure 3:**
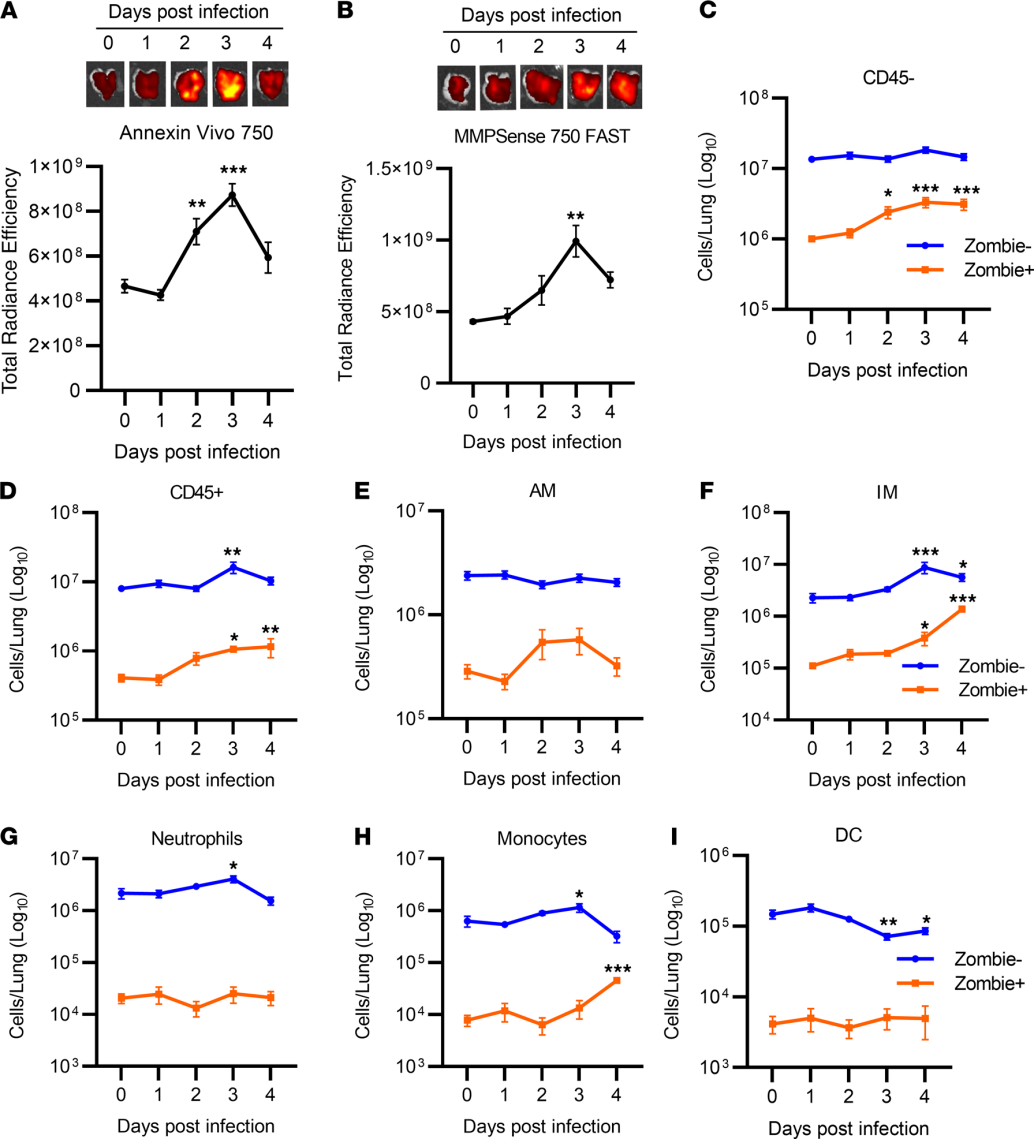
Temporal dynamics of cell death in lung cell populations following infection. (**A** and **B**) Ex vivo imaging of Annexin Vivo 750 (**A**) or MMPSense 750 FAST (**B**) in the lungs of K18-hACE2 mice infected with SARS-CoV-2 (1 × 10^4^ TCID_50_). (**C**–**I**) Flow cytometric analysis of populations of CD45^–^ (**C**) and CD45^+^ (**D**) cells in the lung, alveolar macrophages (AM) (**E**), interstitial macrophages (IM) (**F**), neutrophils (**G**), monocytes (**H**), and dendritic cells (DC) (**I**) including their viability status (Zombie^+^ or Zombie^–^) over time. Data are shown as mean ± SEM (*n* = 8–10 mice pooled from 2 separate experiments). *P < 0.05, **P < 0.01, ***P < 0.001 indicate significance compared with mock-infected control (day 0) using 1-way ANOVA.

The onset of metabolic reprogramming, inflammation, and tissue damage following peak viral burdens may be a consequence of widespread, progressive cell death. To test this assertion, we used in vivo imaging to temporally characterize the cell death and tissue remodeling response following SARS-CoV-2 infection. Infected K18-hACE2 mice were injected i.v. with Annexin Vivo 750, a far-red fluorescent probe that binds exposed phosphatidylserine residues on apoptotic or necrotic cells, or MMPSense 750 FAST for detection of active matrix metalloproteinases (MMPs) as a marker for tissue remodeling. Annexin Vivo 750 fluorescence was significantly increased in the lungs of SARS-CoV-2–infected mice on day 2 after infection relative to mock-infected controls (day 0) and peaked on day 3 after infection (Figure 3A). Additionally, we observed increased MMPSense 750 FAST fluorescence by day 3 after infection relative to uninfected controls (Figure 3B). No significant increase in Annexin Vivo 750 or MMPSense 750 FAST signal was evident in systemic tissues including the kidney, spleen, liver, and brain of infected mice relative to mock-infected controls (data not shown), indicating that pathological processes were primarily restricted to the pulmonary compartment. Collectively, these imaging results suggest that early cell death is a likely trigger for host inflammatory cascades, metabolic stress, and tissue injury.

We next performed flow cytometric analysis of lung cell populations over time, including their viability status, to further characterize the host cell death response during SARS-CoV-2 infection. Isolated whole-lung single-cell populations were phenotypically distinguished as CD45^–^ and CD45^+^ populations, then further delineated by surface markers as alveolar macrophages (SiglecF^+^CD11c^+^), interstitial macrophages (SiglecF^–^CD11c^int^CX3CR1^+^CD11b^+^), neutrophils (SiglecF^–^CX3CR1^–^CD11b^+^Ly6G^+^), dendritic cells (SiglecF^–^CX3CR1^–^Ly6G^–^CD11c^+^CD103^+^), and/or monocytes (SiglecF^–^CX3CR1^–^Ly6G^–^CD11b^+^Ly6C^int^) (see Supplemental Figure 3 for gating strategy). Additionally, cells were stained with the live/dead discriminatory dye Zombie. In agreement with Annexin Vivo 750 fluorescence, we observed a significant increase in CD45^–^Zombie^+^ cells on day 2 after infection that further increased through day 4 after infection (Figure 3C), suggesting increased cell death in nonleukocyte populations. On days 3 and 4 after infection, we observed a significant increase of viable CD45^+^ cells (Figure 3D). Temporally, early cell death results in release of DAMPs including HMGB1, which can have both chemotactic and inflammation-stimulating activity leading to cellular recruitment (33). Furthermore, we observed increased MCP-1 levels on day 2 after infection, suggesting activation of macrophage- and monocyte-specific chemotaxis (Figure 1H). Consistent with this notion, recruited cells primarily consisted of interstitial macrophages with monocyte and marginal neutrophil recruitment (Figure 3, D–I). Interstitial macrophages with marginal neutrophil contribution have been reported following SARS-CoV infection, where recruited macrophages specifically contribute to tissue injury in a type I IFN–dependent manner (34). In parallel with cellular influx of innate leukocyte populations was a progressive increase in Zombie^+^ populations among interstitial macrophages and monocytes (Figure 3, E and H). We also observed a significant reduction in lung dendritic cells following infection, potentially reflecting trafficking to the draining lymph tissue following activation (Figure 3I).

### FPS-ZM1 treatment improves survival in SARS-CoV-2–infected K18-hACE2 mice.

**Figure 4:**
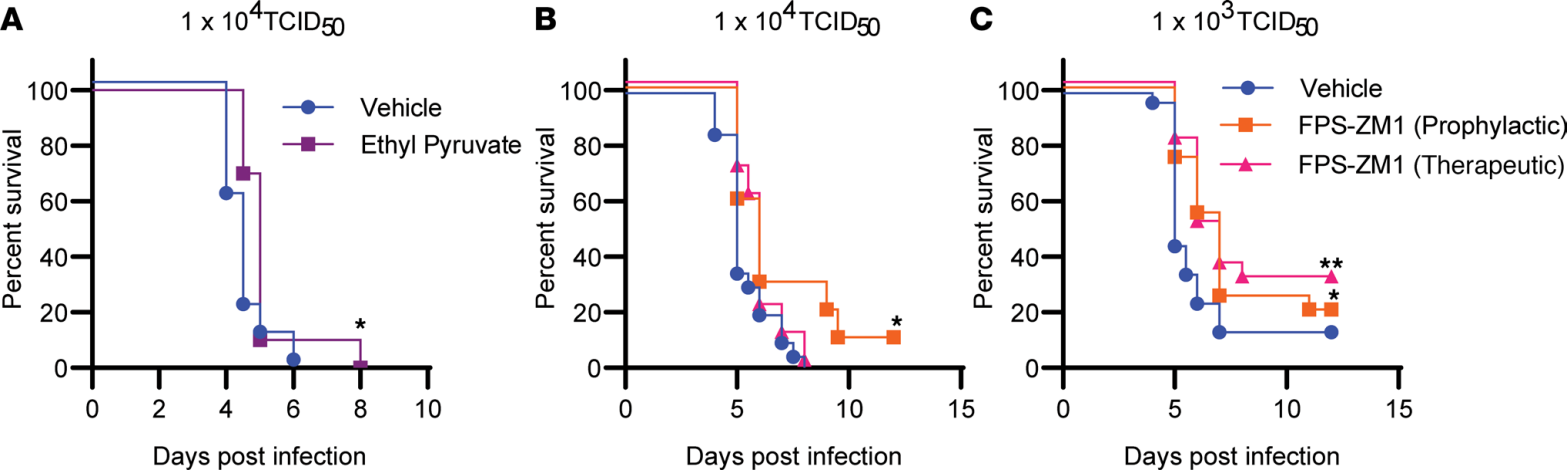
FPS-ZM1 treatment improves survival in SARS-CoV-2–infected mice. K18-hACE2 mice were infected by intranasal instillation with the indicated dose of SARS-CoV-2 and given vehicle, ethyl pyruvate, or FPS-ZM1 daily by i.p. injection. Prophylactic dosing was used for ethyl pyruvate and vehicle groups. Both prophylactic and therapeutic dosing schedules were used for FPS-ZM1 (see Methods for details). (**A**) Survival of mice receiving 1 × 10^4^ TCID_50_ and vehicle or ethyl pyruvate treatment. (**B**) Survival of mice receiving 1 × 10^4^ TCID_50_ and vehicle or FPS-ZM1 treatment. (**C**) Survival of mice receiving 1 × 10^3^ TCID_50_ and vehicle or FPS-ZM1 treatment. Data shown are from *n* = 20 mice per group, pooled from 2 separate experiments. *P < 0.05, **P < 0.01 indicate significance compared with vehicle-treated controls using a log-rank Mantel-Cox test.

Cell death is accompanied by the release of alarmins that can activate RAGE signaling leading to upregulation of inflammation or coagulation cascades. Given evidence of cell death followed by increased glycolysis and production of proinflammatory cytokines described above, we hypothesized that RAGE signaling may be promoting SARS-CoV-2–related disease pathogenesis. We initially tested the impact of the nonspecific RAGE/HMGB1 signaling inhibitor ethyl pyruvate on host survival following a uniformly lethal infection with SARS-CoV-2 (1 × 10^4^ TCID_50_) (35). Among animals treated with ethyl pyruvate, we observed a small, but statistically significant, extension in median time to death of 1 day in comparison with vehicle controls (Figure 4A). We next tested the RAGE-specific inhibitor FPS-ZM1 for its ability to impact SARS-CoV-2–driven mortality. FPS-ZM1 preferentially interacts with the V domain of RAGE and has a reported *K_I_* of 25 ± 5 nM (36). Among animals given a uniformly lethal dose of SARS-CoV-2 (1 × 10^4^ TCID_50_), only animals treated prophylactically with FPS-ZM1 had a significant increase in survival and extended median time to death (Figure 4B). In contrast, in animals infected with a lower dose (1 × 10^3^ TCID_50_, approximately equal to an LD_90_), both prophylactic and therapeutic treatment with FPS-ZM1 significantly increased survival and extended median time to death (Figure 4C). Moreover, although not statistically significant, animals that received FPS-ZM1 therapeutically exhibited greater survival compared with those receiving the drug prophylactically.

### FPS-ZM1 treatment limits the host inflammation following infection.

**Figure 5:**
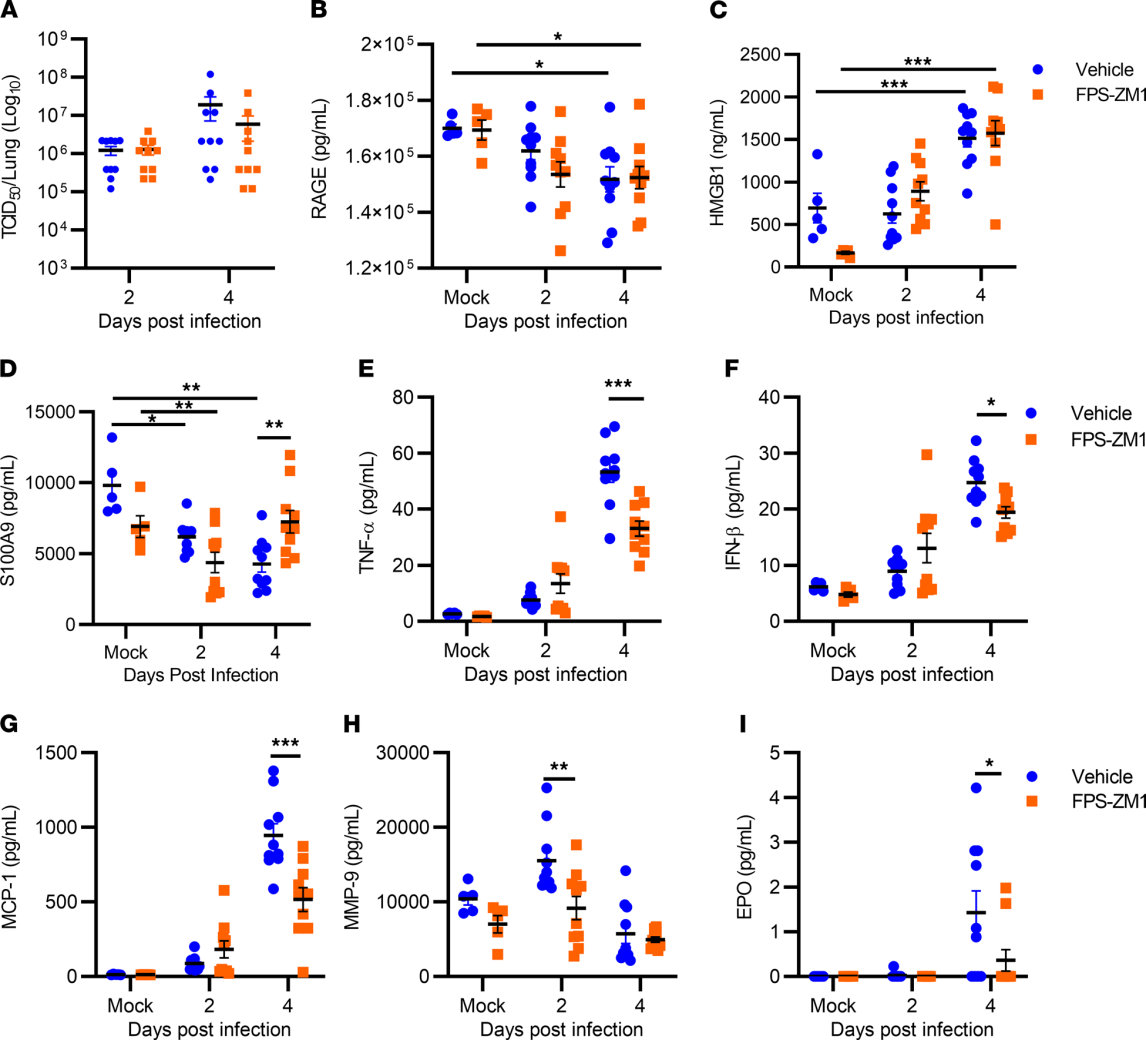
FPS-ZM1 treatment limits the host inflammatory response following infection. K18-hACE2 mice were infected with 1 × 10^3^ TCID_50_ SARS-CoV-2 by intranasal instillation and therapeutically treated with vehicle or FPS-ZM1. (**A**–**D**) Whole-lung homogenates were evaluated for viral burden (**A**), RAGE (**B**), HMGB1 (**C**), and S100A9 levels (**D**). Lung homogenates were also evaluated for 50 additional proinflammatory cytokines/chemokines (see Supplemental Table 1). (**E**–**I**) Several important mediators known for their critical role in lung injury were selectively presented from that array, including TNF-α (**E**), IFN-β (**F**), MCP-1 (**G**), MMP-9 (**H**), and EPO (**I**). Data are shown as mean ± SEM (*n* = 5 for mock-infected, *n* = 10 mice per infected group, pooled from 2 representative experiments). *P < 0.05, **P < 0.01, ***P < 0.001 indicate significance compared with mock-infected, vehicle-treated control, or between vehicle and FPS-ZM1 treatment where indicated, by 2-way ANOVA.

We next investigated the mechanism by which FPS-ZM1 improved survival. Specifically, we assessed whether FPS-ZM1 impacted viral replication in vivo, or whether its effects were primarily due to inhibition of specific tissue inflammation contributing to tissue damage. For these experiments, mice were infected with 1 × 10^3^ TCID_50_ of SARS-CoV-2 and were therapeutically treated with FPS-ZM1 or vehicle control as described above. No significant differences were observed in viral burden in the lungs between FPS-ZM1– and vehicle-treated mice (Figure 5A), suggesting that FPS-ZM1 treatment was not affecting survival through antiviral mechanisms. We next assessed whether FPS-ZM1 treatment was impacting host RAGE or DAMP levels or associated downstream cytokine production. Surprisingly, infection resulted in significantly decreased RAGE levels in the lungs on day 4 after infection relative to their respective mock-infected and vehicle- or FPS-ZM1–treated controls (Figure 5B). No significant difference in RAGE levels was observed between infected mice treated with vehicle versus FPS-ZM1. In contrast, the RAGE agonist HMGB1 was significantly elevated in the lungs on day 4 after infection relative to the appropriate uninfected control (Figure 5C). FPS-ZM1 treatment reduced baseline levels of HMGB1 in the lungs but did not alter the trajectory of increased HMGB1 levels as infection progressed. We observed a time-dependent decrease in levels of another RAGE agonist, S100A9, in the lungs of infected mice treated with vehicle (Figure 5D). FPS-ZM1 treatment maintained S100A9 levels in the lung on day 4 after infection relative to vehicle-treated mice (Figure 5D). We also observed a time-dependent increase in most inflammatory cytokines/chemokines among vehicle-treated groups (representative cytokines/chemokines shown in Figure 5, E–H; see also Supplemental Table 1). In agreement with our imaging studies, MMP-9 was significantly elevated on day 2 after infection in vehicle-treated mice compared with mock-infected controls (Figure 5H). Furthermore, there was a significant increase in erythropoietin (EPO) in the lung on day 4 after infection (Figure 5I). Increased EPO may reflect the host response to curb hypoxia by increasing the systemic oxygen-carrying capacity (37). As predicted, FPS-ZM1 treatment reduced the level of the majority of proinflammatory cytokines/chemokines in lungs on day 4 after infection (Figure 5, E–G, and Supplemental Table 1). FPS-ZM1 treatment also reduced levels of MMP-9 on day 2 after infection and EPO on day 4 after infection (Figure 5, H and I).

### FPS-ZM1 treatment reduces lung perivascular inflammation.

**Figure 6:**
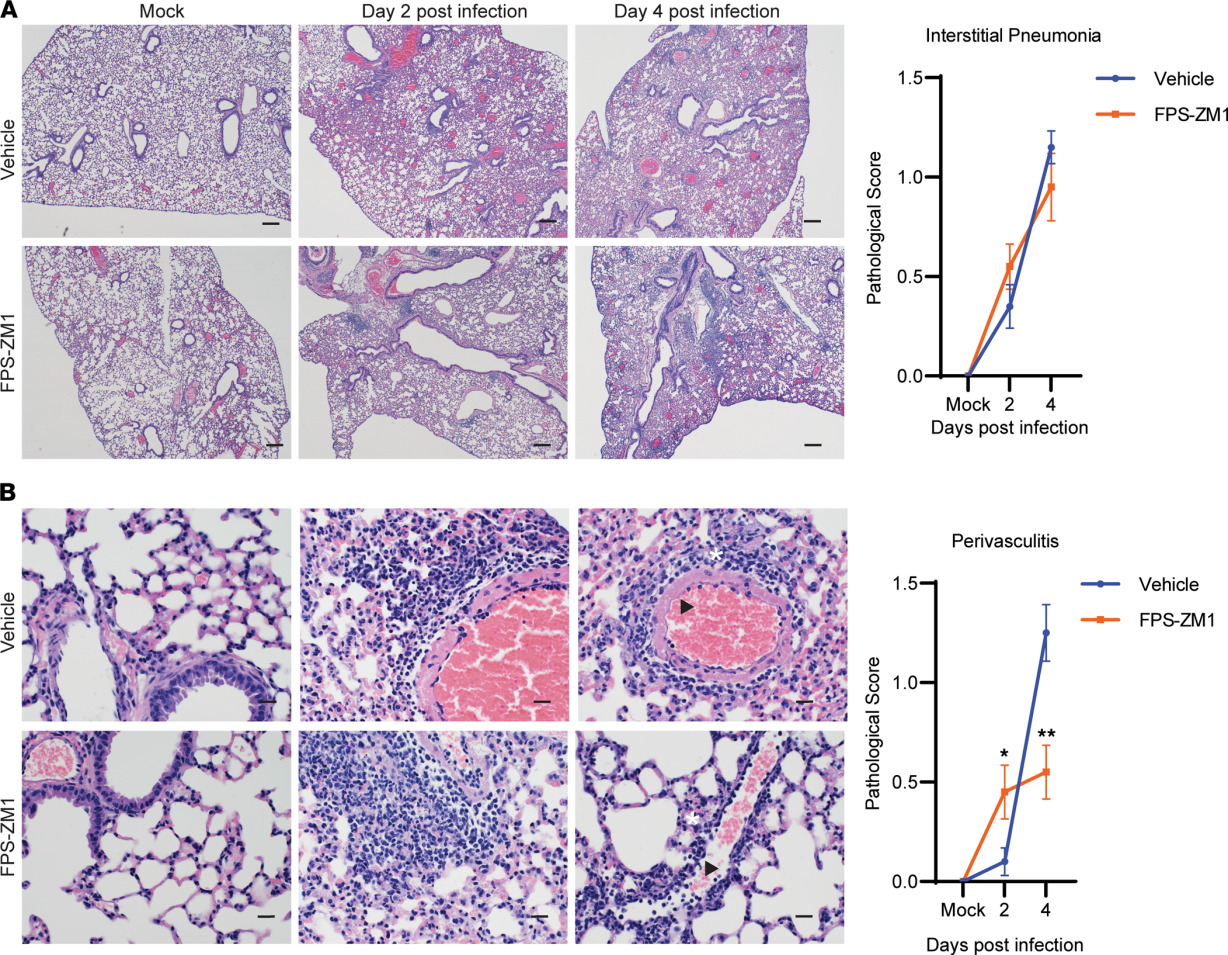
FPS-ZM1 treatment reduces lung perivascular inflammation. K18-hACE2 mice were infected with 1 × 10^3^ TCID_50_ SARS-CoV-2 by intranasal instillation, therapeutically treated with vehicle or FPS-ZM1, and evaluated for changes in histopathology. Lesions were scored between 0 (no lesion) and 5 (severe). (**A**) Representative figures of lungs from mock- or SARS-CoV-2–infected mice and vehicle or FPS-ZM1 treatment on days 2 and 4 after infection along with corresponding pathological scores. (**B**) Representative images of perivascular cuffing along with pathological scores (×400). The white asterisk indicates regions of perivascular inflammation and interstitial thickening that are reduced in mice with FPS-ZM1 treatment compared with vehicle-treated controls. Black arrowheads show select regions in which hypertrophy of the endothelial cells in the vessel wall is reduced in FPS-ZM1–treated mice. (×40 and ×400 magnification; scale bars: 20 μm at ×400, 200 μm at ×40.) Data are shown as mean score ± SEM (*n* = 20 per group, pooled from 2 separate experiments). *P < 0.05, **P < 0.01 indicate significance between vehicle and FPS-ZM1 treatment using an unpaired, nonparametric Mann-Whitney test.

Given the marked reduction in inflammatory cytokines within the lung following FPS-ZM1 treatment on day 4 after infection, we hypothesized that this would correlate with attenuated lung pathology. Therefore, we compared pathological changes in the lung on days 2 and 4 after infection following FPS-ZM1 treatment relative to vehicle-treated controls. The overall character of the pathological changes in lung architecture was similar between vehicle and FPS-ZM1 treatment (Figure 6, A and B). While there was no significant impact of FPS-ZM1 treatment on the extent of interstitial pneumonia (Figure 6A), a modest, but significant, reduction in perivasculitis was observed among FPS-ZM1–treated mice (Figure 6B). Together, these results confirm that RAGE inhibition by FPS-ZM1 treatment contributes to limiting host inflammation and specifically reduces pathology in the perivasculature regions of the lung leading to increased survival following lethal SARS-CoV-2 infection.

## Discussion

One of the challenges of therapeutic intervention among individuals infected with SARS-CoV-2 is the limited understanding of the temporal nature of the molecular and cellular events driving disease progression. Current antiviral or antiinflammatory treatments can have limited efficacy if administered outside therapeutic windows associated with these distinct events. Herein, using the K18-hACE2/SARS-CoV-2 mouse model of infection, we demonstrate that there are at least 2 distinct phases of disease pathogenesis based on molecular events. Initially we found SARS-CoV-2 replicated to high titers in the lungs without facilitating host metabolic reprogramming or tissue damage. This phase was followed by a cell death event in nonmyeloid populations leading to progressive tissue damage and inflammation, even as viral burdens decrease. These data suggest that a more tailored therapeutic approach may be required for optimal treatment of SARS-CoV-2 infection.

Considering our findings showing distinct phases of infection, strategies that limit viral replication may be most efficacious during early stages of infection before evidence of widespread cell death. This claim is supported by studies in nonhuman primates using SARS-CoV-2 or MERS-CoV infection, which have reported decreased lung disease only when the antiviral remdesivir was administered prophylactically or prior to peak viral burden (38, 39). Similarly, remdesivir marginally impacted mortality rates in humans hospitalized with SARS-CoV-2, a likely consequence of these patients being outside of the early phase of viral replication (40). Consistent with this notion, as well as the established inhibitory activity of FPS-ZM1 against RAGE (36), we did not expect treatment with this drug to directly limit viral replication as a mechanism to reduce lung injury. Indeed, viral loads in mice treated with FPS-ZM1 were not different from those in vehicle-treated animals infected with SARS-CoV-2. However, at later stages of infection when lung injury is primarily driven by cell death and dysregulation of host inflammation, the data herein support that inhibiting RAGE could be an effective therapeutic strategy.

Cell death can provide a major source of DAMPs needed to activate RAGE signaling cascades. Characterization of the cell death response following lethal SARS-CoV-2 infection indicated early cell death among CD45^–^ populations that could provide an initial flux of DAMPs. While the specific CD45^–^ subset(s) undergoing death were not defined in these studies, lung pneumocytes are reportedly primary impacted populations (1, 41). HMGB1 was specifically elevated in the lungs of SARS-CoV-2–infected mice, consistent with an increased DAMP response. FPS-ZM1 treatment did not reduce DAMP levels in the lung, suggesting that this treatment may not directly impact early cell death. However, cell death in both the CD45^+^ and CD45^–^ populations steadily increased as the infection progressed, and FPS-ZM1 treatment may limit inflammation and associated tissue damage resulting from cell death at these later stages of infection. Further studies are needed to specifically determine the impacts of FPS-ZM1 treatment on cell death in vivo.

A secondary consequence of cell death is the upregulation MMPs, which can remodel tissue and drive compromised lung function (42). Clinically, MMP-9 levels have shown strong correlation with respiratory failure in SARS-CoV-2–infected patients (43). Broad MMP inhibitors have been shown to improve outcomes in animal models of lung injury and have been proposed as therapeutics for SARS-CoV-2–infected patients (42). RAGE inhibition via FPS-ZM1 corresponded with a decrease in lung MMP-9 levels in the current study. It is possible that reduction in MMP-9 levels with FPS-ZM1 treatment may limit exuberant tissue remodeling or damage following cell death. However, genetic depletion of MMP-9 has provided conflicting evidence showing both positive and negative roles in lung injury depending on the etiology (42). Therefore, further interrogation of the contribution of RAGE to regulation of other MMP levels and activity is warranted.

Another potential mechanism by which RAGE inhibition may reduce tissue injury is through impairment of type I IFN responses via IRF-7. Evidence of a RAGE/IRF-7 signaling pathway has been reported (44). Perlman and colleagues have demonstrated that delayed type I IFN responses contribute to severe disease pathogenesis during SARS-CoV infection whereas absence of a type I IFN response results in minimal preclinical disease (34). We observed a significant reduction in IFN-α and IFN-β with FPS-ZM1 treatment, potentially contributing to reduced pathology and increased survival. However, whether reduction in IFN-α or IFN-β levels was due to FPS-ZM1 inhibition of RAGE/IRF-7 signaling or a consequence of interruption of broader RAGE signaling responses is not clear. The reduction in multiple cytokine and chemokines families by FPS-ZM1 suggests a broad immunomodulatory effect that may also limit IRF-7 activation. Further studies are needed to confirm this supposition.

RAGE signaling is also known to play a key role in regulating the renin-angiotensin system (RAS). RAGE has been reported to be protective in models of kidney and cardiac injury caused by hypertension through downregulation of oxidative stress (45). Clinically, dysregulation of the RAS is evident in critically ill SARS-CoV-2–infected patients (46). Furthermore, many patients suffer from hypertension and other vascular comorbidities linked to dysregulation of the RAS and exacerbation of disease (18, 19, 47, 48). Coincidently, SARS-CoV-2 uses ACE2, a key receptor within the RAS, to invade cells. Following infection, ACE2 is downregulated, which may reduce its established tissue-protective functions (49). Direct interaction between RAGE and ACE2 has been documented, but the extent of interaction has not been fully elucidated. For example, a ligand-independent mechanism for RAGE transactivation has been identified via angiotensin II that results in activation of host inflammatory pathways irrespective of the presence of DAMPs (50). Hypothetically, this could be a mechanism by which FPS-ZM1 reduces inflammation and tissue damage. However, whether RAGE and ACE2 colocalization occurs in the lung following infection allowing engagement of this response is not known. Alternatively, targeting RAGE may reduce inflammation, oxidative stress, and tissue injury typically activated upon detection of DAMPs, leading to less dysregulation of the RAS.

Current available animal models present a broad spectrum of disease in response to SARS-CoV-2 infection. K18-hACE2 mice have received extensive attention due to their high susceptibility to infection. Several studies have indicated that the K18-hACE2 mouse recapitulates pathological features in the lung consistent with severe clinical disease, including extensive interstitial pneumonia, perivasculitis, edema, and microthrombosis (20, 21, 51). Evidence has also been reported of brain involvement (23). Characterization of this model in the current study revealed only minimal to mild interstitial pneumonia and perivasculitis with minimal or no evidence of airway edema, hyaline membrane formation, congestion, or vascular thrombosis, despite up to 80% of mice becoming moribund by day 4.5 after infection when given 1 × 10^4^ TCID_50_ SARS-CoV-2. Further, there were no pathological changes in systemic tissues, including the brain, that could have provided a significant contribution to morbidity. Proinflammatory cytokine levels and viral burdens peaked between days 2 and 3 after infection, indicating that general lung inflammation and viral load were not driving morbidity. However, this finding does not exclude the possibility that specific tissue damage is occurring associated with inflammation and leading to compromised lung function. We observed decreased oxygen saturation associated with increased cell death in lung CD45^–^ cell populations, indicating that gas exchange is indeed compromised despite the rarity of airway edema or alveolar infiltrates. Impaired gas exchange can also occur in response to damaged pulmonary microvasculature. Typically, hypoxemia associated with prototypical acute respiratory distress syndrome (ARDS) results from airway consolidation or atelectasis and reduced lung compliance. However, Chiumello and colleagues reported an atypical ARDS manifestation in SARS-CoV-2–infected patients that included severe hypoxemia with preserved lung compliance (26). These data suggest a strong vasculature component driving systemic hypoxemia in critically ill patients infected with SARS-CoV-2. Vascular injury and coagulation dysregulation during SARS-CoV-2 infection have been repeatedly documented in the clinic and are in line with deregulation of the RAS (27). Supportively, recent reports demonstrate endothelial cell injury and thrombosis in the K18-hACE2 mouse model (21). In the current study, one of the most prominent pathological features evident in SARS-CoV-2–infected K18-hACE2 mice was perivascular cuffing including edema and endothelial hypertrophy. Perivascular pathology was modestly reduced with FPS-ZM1 treatment, indicating an important role for RAGE in driving injury to the pulmonary vasculature. We did not find any evidence of thrombosis. While the specific contribution of perivasculitis to systemic hypoxia was not investigated, it is tempting to speculate based on the effects of FPS-ZM1 that vasculature dysregulation following increased cell death in CD45^–^ cell populations, including type I and II pneumocytes and/or endothelial cells, may contribute to the rapid decline of K18-hACE2 mice between days 4 and 6 after infection.

Herein, we present a map of SARS-CoV-2 pathogenesis in the K18-hACE2 mouse model that revealed RAGE signaling as a promising therapeutic target to limit damaging host responses. Optimization of FPS-ZM1 dosing and bioavailability in the lungs was not determined in the current study but may further bolster the positive effect of this drug for treating SARS-CoV-2 infection. Additionally, how late during infection FPS-ZM1 could be administered and provide a survival benefit is not known and is currently under investigation. Regardless, the data herein support expansion of preclinical testing of FPS-ZM1 in additional animal models of SARS-CoV-2 and provide proof-of-principle justification for greater interrogation of RAGE signaling in humans during severe SARS-CoV-2 infection.

## Methods

### SARS-CoV-2.

SARS-CoV-2 (USA/WA1/2020) was obtained from BEI Resources and propagated in Vero cells to generate viral stocks. Stock solutions were frozen at –80°C until use.

### Mice.

B6.Cg-Tg(K18-ACE2)2Prlmn/J mice (K18-hACE2) of mixed sexes were purchased from The Jackson Laboratory and/or bred in-house. All mice were specific pathogen free and were housed in animal biosafety level 3 facilities at Rocky Mountain Laboratories. K18-hACE2 mice were used between 6 and 12 weeks of age, housed on a 12-hour light/12-hour dark cycle, and provided with food and water ad libitum.

### Intranasal infection with SARS-CoV-2.

Mice were anesthetized with ketamine/xylazine, and 25 μL of SARS-CoV-2 inoculum diluted in PBS was delivered by intranasal instillation in the left naris. Mice receiving PBS alone served as negative controls. FPS-ZM1 (EMD Millipore) was dissolved in DMSO and diluted in PBS before injection in mice. For prophylactic dosing of FPS-ZM1, mice were injected with FPS-ZM1 (1 mg/kg) or vehicle (PBS plus DMSO) via the i.p. route 24 hours before infection, 8 hours after infection, then every 24 hours thereafter for the duration of the study. For therapeutic intervention, mice were injected with FPS-ZM1 8 hours after infection, then every 24 hours thereafter for the duration of the study. Mock-infected controls were harvested on day 2 after infection following the dosing regimen outlined above.

### Histology.

Lungs were removed and fixed in 10% neutral-buffered formalin with 2 changes of formalin for a minimum of 7 days prior to processing. Lung tissue was processed with a Sakura Tissue-Tek VIP-5 processor on a 12-hour automated schedule using a graded series of ethanol, xylene, and Paraplast Xtra (MilliporeSigma). Paraffin-embedded tissues were sectioned at 5 μm thickness and dried overnight at 42°C before staining. Fixed tissue sections were stained with H&E or for SARS-CoV-2 N antigen using GenScript U864YFA140-4/CB2093 NP-1 diluted 1:1000 with an anti-rabbit IgG polymer (Vector Laboratories ImPress VR). Tissues were processed for IHC using a Discovery Ultra automated processor (Ventana Medical Systems) with ChromoMap DAB Kit (Roche Tissue Diagnostics). Stained slides were examined on an Olympus BX51 light microscope equipped with an Olympus DP722 camera and associated cellSens Dimension 1.4.1 software. Pathological analysis was performed blinded by a board-certified pathologist. Lesions were scored from 0 (no lesions) to 5 (severe).

### Pulse oximetry.

On days 4–5 after infection, mice were anesthetized with a ketamine/xylazine cocktail and the right hind limb shaved to allow for penetration of infrared light. A thigh sensor (STARR Life Sciences Corp.) was then placed on the hind limb and oxygen saturation readings acquired over a duration of 30–180 seconds using a MouseOx Plus Pulse Oximeter.

### Tissue processing for cytokine and viral burden quantification.

Lungs were aseptically removed and placed in cold complete DMEM (cDMEM; 5% FBS, l-glutamate, sodium pyruvate, NEAA, and HEPES). Tissues were homogenized by grinding through a sterile S/S type 304 no. 60 wire mesh screen (Belleville Wire Cloth Co.) using a 5 mL syringe plunger. Tissues were then centrifuged at 9400*g* for 5 minutes, and supernatant was collected and frozen at –80°C until downstream analysis.

### Quantification of viral burden.

Viral titer was determined by TCID_50_ assay. Vero cells were plated at 1 × 10^4^ per well in a flat-bottom 96-well plate in cDMEM. After 24 hours, Vero cells were infected with samples serially diluted in cDMEM and then incubated for 1 hour at 37°C with 5% CO_2_. Samples were removed and fresh cDMEM added to the infected Vero cultures. Plates were incubated for 72 hours after infection and evaluated for cellular lysis and well clearance. Briefly, supernatant was removed, and cells were fixed with 100 μL of 4% formalin for 1 hour at room temperature. Formalin was removed and cells washed once with PBS, and then 25 μL of 0.1% crystal violet stain in methanol was added to each well. Cells were incubated for an additional 15 minutes; then stain was removed, and cells were washed twice with distilled water. TCID_50_ was calculated according to the Reed-Muench method.

### Inflammatory cytokine, RAGE, and DAMP quantification.

Inflammatory cytokines/chemokines were quantified in whole-lung homogenates using a Meso Scale multiplex cytokine array following the manufacturer’s instructions (U-Plex Biomarker Group 1, Meso Scale). RAGE and the RAGE ligand S100A9 were quantified in whole-lung homogenates collected from mice using the RAGE Quantikine ELISA Kit and Mouse S100A9 DuoSet ELISA Kit, respectively (R&D Systems). HMGB1 was quantified in whole-lung homogenates and mouse serum by ELISA (HMGB1 Express, Tecan US).

### Ex vivo imaging.

For studies imaging XenoLight RediJect 2-DG and Annexin Vivo 750, 2 hours after i.v. injection was found to be the optimal time point to image maximal signal and tissue distribution (data not shown), consistent with the manufacturer’s recommendations (PerkinElmer). Total MMP activity was assessed using MMPSense 750 FAST, a probe that becomes fluorescently active upon cleavage by matrix metalloproteinases. Mouse tissues were imaged 6 hours after injection of MMPSense 750 FAST according to the manufacturer’s recommendations (PerkinElmer). All probes were injected in 100 μL via the retro-orbital sinus. At the indicated imaging times, mice were euthanized by isoflurane inhalation and whole organs aseptically removed and placed in a 90-mm-diameter Petri dish. Tissues were immediately imaged with an IVIS Lumina XR imaging system using the ICG filter. Images were analyzed using Living Image 4.0 (PerkinElmer). Regions of interest were drawn around the excised tissue, fluorescent signal intensity measured, and total radiance efficiency calculated. Background autofluorescence was corrected for using tissues from mice in which no fluorescent probe was injected.

### Mass spectrometry.

For all liquid chromatography–mass spectrometry (LC-MS) analyses, LC-MS–grade solvents and reagents were used. Left lung lobes of the mice were collected into 500 μL of ice-cold methanol. Organs were subsequently homogenized, and 500 μL of water was added. To the suspension 500 μL of chloroform was added, and the sample was agitated via shaking at 4°C for 20 minutes. Layering was induced by centrifugation at 16,000*g*, 4°C for 20 minutes. The polar (upper) layer was collected and diluted 1:10 in 50% methanol in water. Analysis was performed consistent with previous reports (52). Samples were injected onto a Sciex ExionLC AC system and separated using an ion pairing strategy on a Waters Atlantis T3 column (100 Å, 3 μm, 3 mm × 100 mm) with a 15-minute gradient from 5 mM tributylamine, 5 mM acetic acid in 2% isopropanol, 5% methanol, 93% water (vol/vol) to 100% isopropanol. Metabolites were detected on a Sciex 5500 QTRAP mass spectrometer using a MRM strategy consistent with previous reports (53). QC injections were performed after every 10 injections to ensure instrument stability. All data were processed and filtered using MultiQuant Software 3.0.3 (Sciex). A 50% missing value cutoff and 30% QC coefficient of variance cutoff were imposed. Remaining missing values were replaced with the minimum group value for that feature. Following filtering, the data set was total sum normalized. All univariate and multivariate analysis was performed in MarkerView Software 1.3.1 (Sciex).

### Flow cytometry.

Lungs were aseptically removed and placed in ice-cold PBS before processing for flow cytometry. Lungs were then minced and digested using Liberase TM (Sigma-Aldrich) as described previously (54). ACK lysis buffer was used to lyse the red blood cells, and then total number of viable cells enumerated using trypan blue exclusion. Cell counts were obtained using a TC20 automated cell counter (Bio-Rad). Single-cell suspensions were then stained with Zombie Near-IR (BioLegend) to determine live and dead cells and with the following antibodies for characterization of innate immune populations: CD45 BUV385, CD11b BV510, CD11c–PerCP-Cy5.5, CX3CR1 BV785, SiglecF-PE, CD103 BV711, Ly6C-APC, Ly6G AF700 (BioLegend). Data acquisition was conducted on a Symphony Flow Cytometer (BD Biosciences) and data analyzed using FlowJo 10 (BD Biosciences). The gating scheme used for determining lung cell populations is provided in Supplemental Figure 3.

### Statistics.

Statistical significance between group means was determined using 1- or 2-way ANOVA, followed by Tukey’s or Dunnett’s multicomparison tests to compensate for type I error. An unpaired, nonparametric Mann-Whitney test was used for comparisons of histopathological scores. A log-rank Mantel-Cox test was used to evaluate significance within survival studies between vehicle- and FPS-ZM1–treated groups. Statistical power was greater than <0.8 to determine sample size, and statistical significance was defined as a probability of type I error occurring at *P* less than 0.05. All studies were repeated twice with 5–10 mice per group for each experimental replicate. Data were pooled (10–20 mice total) for statistical analysis. All statistical analysis was performed using GraphPad Prism 9.0.

### Study approval.

All studies involving K18-hACE2 mice were done with the approval of and in accordance with the Animal Care and Use Committee at Rocky Mountain Laboratories (Animal Study Protocol 2020-70-E).

## Author contributions

FJ, JRH, and CMB conceptualized the studies. FJ, LMR, BS, and EB developed the methods and performed the experiments. JRH provided resources. DS performed the histological evaluations, tissue section imaging, and pathological scoring. FJ, LMR, and BS wrote the original draft. FJ, LMR, BS, JRH, and CMB reviewed and edited the manuscript.

## Supplementary Material

Supplemental data

## Version 1 01/25/2022

Electronic publication
