# Supplementary material for: Impairing RAGE signaling promotes survival and limits disease pathogenesis following SARS-CoV-2 infection in mice
Source: JCI Insight. 2022 Jan 25;7(2):e155896. doi: 10.1172/jci.insight.155896 (PMC8855831; doi:10.1172/jci.insight.155896)
Supplement: Supplemental data [file jciinsight-7-155896-s205.pdf]

Supplemental Figure 1

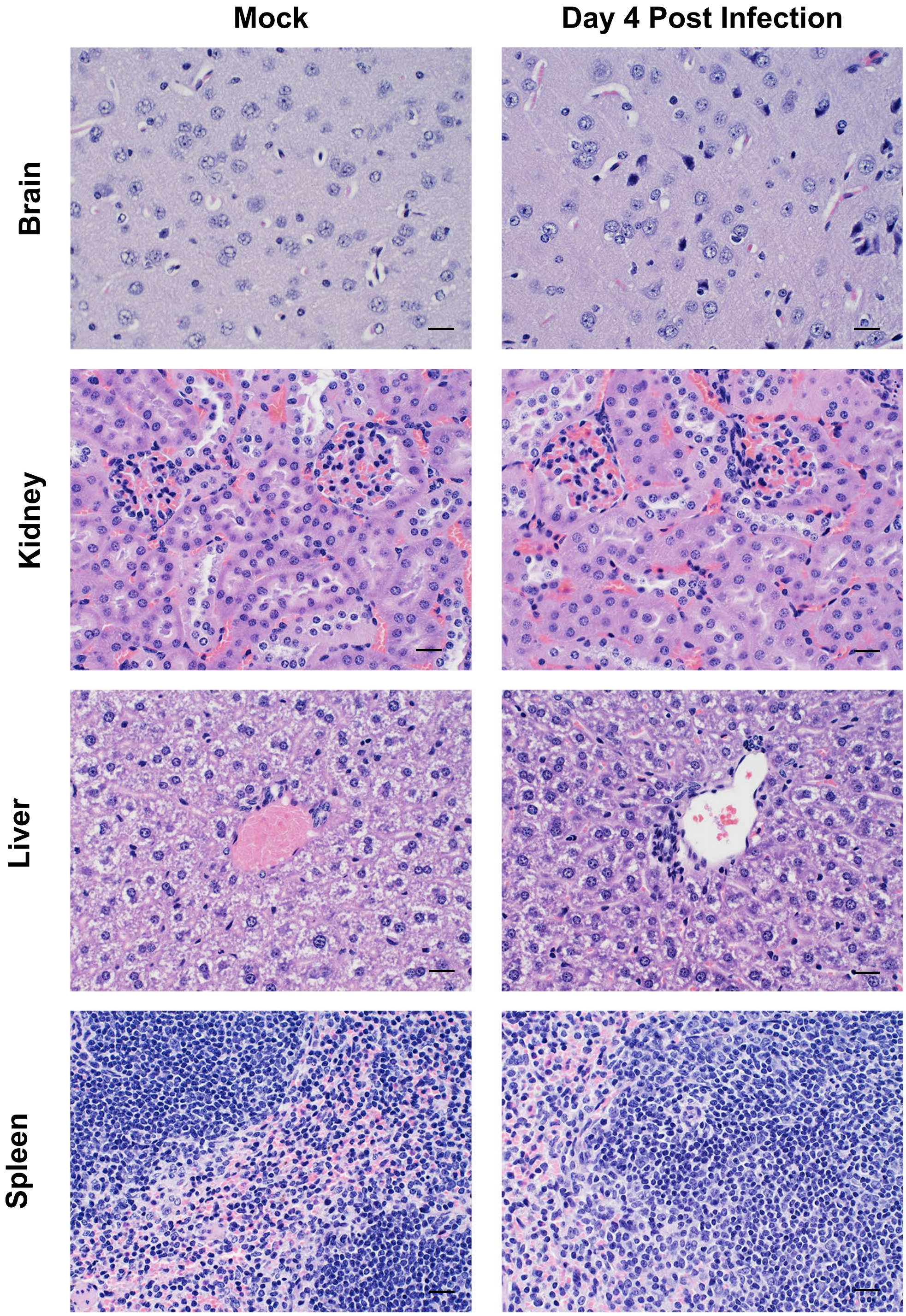

Supplemental Figure 2

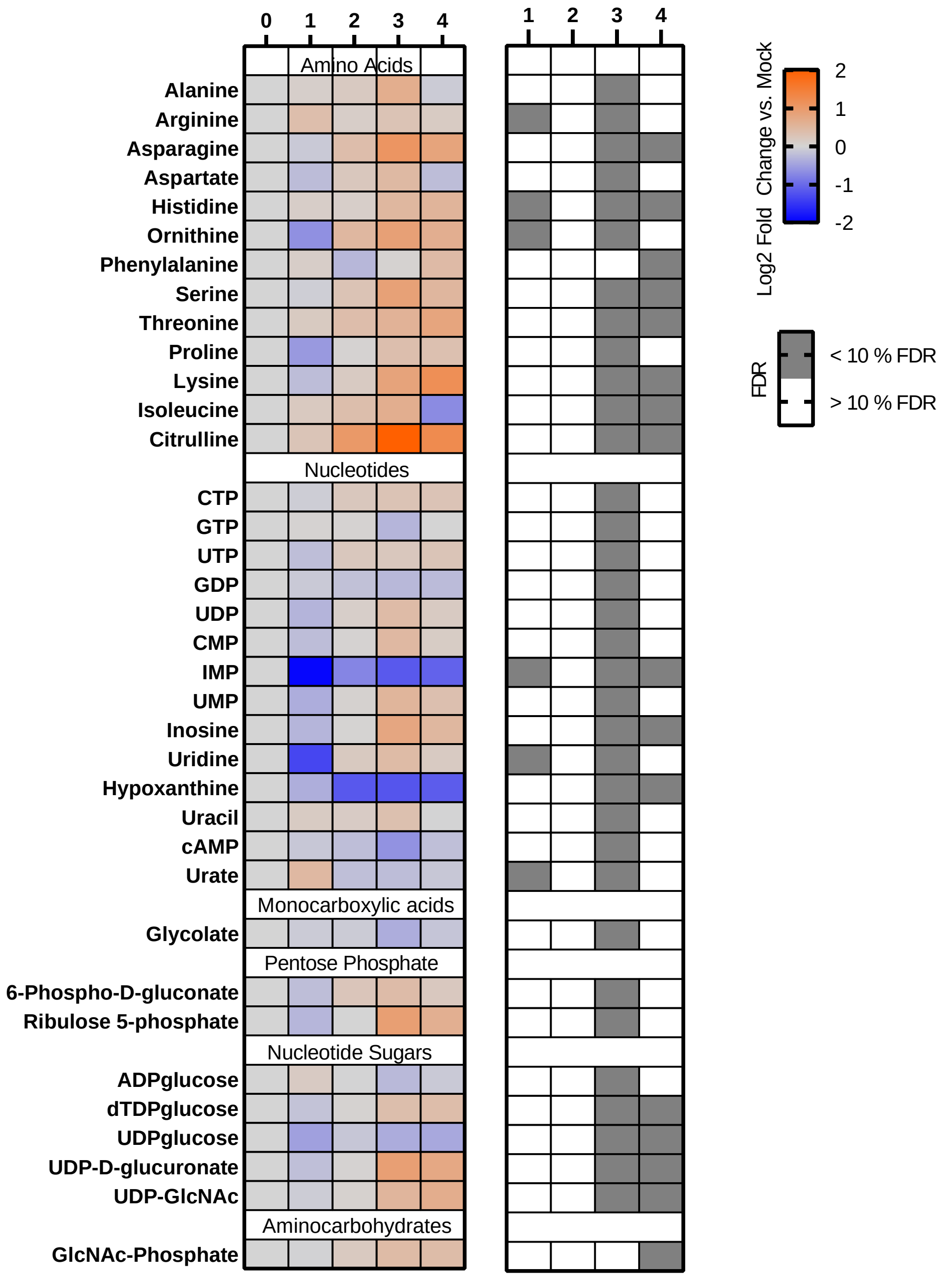

Supplemental Figure 3

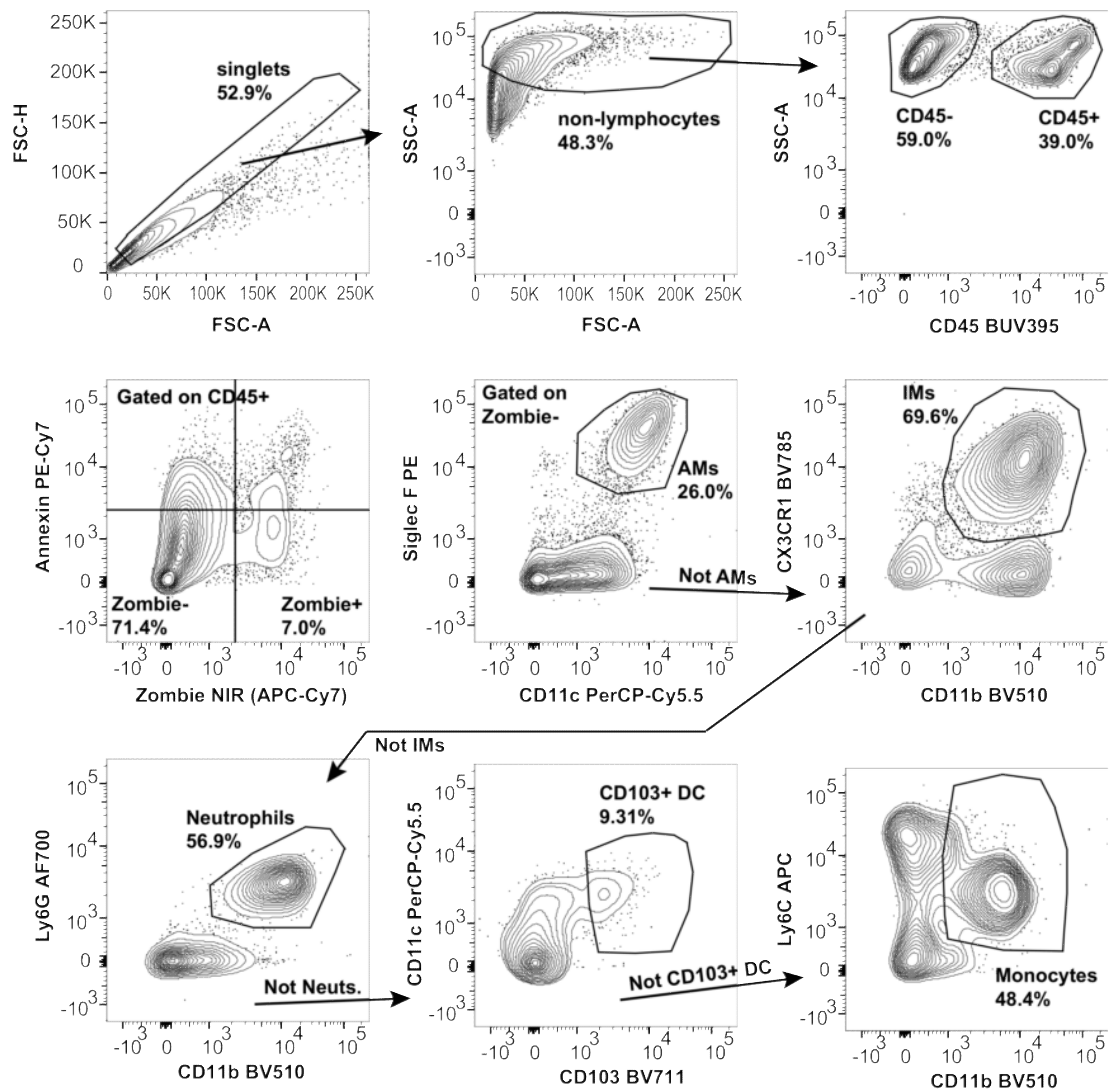

Supplemental Figure 1: Characterization of SARS-CoV-2 disease pathogenesis. Representative images of brain, kidney, liver, and spleen tissue from moribund K18-hACE2 mice on day 4 post-infection relative to uninfected controls. Images are shown at 400X magnification (Black bar = 20 µm). Images are representative of 2 separate experiments with n= 10 mice per group.

Supplemental Figure 2: SARS-CoV-2 infection drives dysregulation in multiple pathways including amino acid and hypoxanthine metabolism. Semi-targeted analysis of soluble metabolites in the lungs of SARS-CoV-2 infected K18-hACE2 mice over time. Data is shown as the mean of the log_2 Fold Change values relative to the mock control (N=5 mice), and whether the corresponding False Discovery Rate was greater or less than 10%.

Supplemental Figure 3: Flow Cytometry Gating strategy. Representative gating scheme from a day 4 lung sample. After gating on $CD45^+$ cells, $Zombie^-$ and $Zombie^+$ populations were identified. Subsequent gates for individual myeloid populations are shown for the $Zombie^-$ gate to demonstrate how gates were drawn. The Boolean logic tool “Not Gate” within FlowJo was used as indicated by the arrows. The same gates were copied onto the $Zombie^+$ population. Only the $Zombie^-$ populations are shown due to the number of events within all gates.

Supplemental Table 1: Cytokine/Chemokine Array on Lungs from uninfected and SARS-CoV-2 infected mice treated with or without FPS-ZM1

	Vehicle			FPS-ZM1		
	Uninfected	Day 2 post infection	Day 4 post infection	Uninfected	Day 2 post infection	Day 4 post infection
**TH-1 Pro-inflammatory Cytokines**						
**TNF-α**	2.71±0.13	7.58±0.74	53.28±3.67***	1.74±0.11	13.49±3.49	33.10±2.68***^{bbb}
**IL-6**	21.67±2.21	33.13±2.01	132.90±7.95***	18.70±1.03	41.65±8.27	91.14±13.19***^{bb}
**IL-5**	0.20±0.09	7.92±1.01	73.11±7.74***	0.67±0.23	15.71±4.60	47.59±6.67***^{bb}
**IL-2**	83.57±7.86	127.83±9.25	534.54±52.01***	82.95±6.80	145.96±25.91	405.47±38.62***^{b}
**IL-1β**	0.14±0.09	0.39±0.07	1.24±0.11***	0.17±0.03	0.27±0.05	0.94±0.08***^{b}
**IL-12p70**	B.D	0.39±0.21	4.70±0.70***	B.D	1.55±0.39^{a}	4.36±0.90***
**IFN-γ**	0.11±0.03	2.12±0.44	17.49±2.03***	0.05±0.02	4.67±1.74	10.12±1.05***^{bbb}
**IFN-α**	10644.85±495.13	8535.31±958.08	9646.37±1827.23	9830.68±291.29	6602.54±1383.98	5322.86±470.30*^{b}
**IFN-β**	6.16±0.31	8.93±0.82	24.73±1.33***	4.79±0.46	13.06±2.60*	19.43±1.03***^{b}
**IL-12p40**	254.74±8.80	475.70±35.31	1914.12±170.01***	232.55±24.47	507.04±65.55	1286.94±70.78***^{bbb}
**IL-17A**	13.08±1.14	14.58±2.01	18.71±2.33	9.71±0.85	14.68±1.93	18.69±1.75
**IL-17C**	15.56±1.06	20.29±3.24	43.82±7.11	30.11±16.33	21.28±3.14	46.74±10.80
**IL-17E**	15.65±1.14	13.21±1.65	14.22±1.15	7.35±0.92	10.97±1.14	17.46±1.06
**IL-17A/F**	0.66±0.10	1.74±0.32	7.26±0.39***	0.42±0.08	2.15±0.63	4.19±0.62***^{bbb}
**CD40**	319.59±11.34	360.04±45.28	2130.99±217.13***	288.05±31.89	376.40±56.78	940.08±128.08*^{bbb}
**IL-16**	17774.59±608.30	14731.87±491.58*	15044.16±402.96*	16992.54±715.31	13106.20±832.84***	13974.31±713.83**
**MMP-9**	10413.49±833.93	15550.17±1431.35*	5742.51±1295.23	7019.63±1159.83	9166.49±1565.63^{aa}	4945.51±357.08*
**TNF-R1**	175.06±32.93	238.63±33.24	414.03±52.90**	108.33±19.41	201.20±49.95	288.89±25.52^{b}
**NGAL**	192920.60±15342.63	268320.50±39674.54	249663.50±47605.87	721420.50±394803.19	278367.80±28676.95	106973.80±20375.09^{b}
**Chemokines**						

**MCP-5**	15.65±0.62	94.42±9.78*	305.32±21.24***	13.47±0.39	102.64±18.07*	188.19±13.13***[b]
**MDC**	402.40±7.73	598.18±54.49*	503.28±36.56	290.35±27.57	555.00±59.77	470.02±20.43
**BCA-1**	2296.18±102.93	2355.22±151.59	2997.14±194.88*	2312.61±126.01	2204.99±201.61	2266.91±82.77[bb]
**IP-10**	88.03±4.93	3062.19±412.51**	12578.56±812.67***	74.01±74.01	5855.37±5855.37	7076.24±7076.24***[bbb]
**KC**	0.24±0.24	0.82±0.82	2.14±2.14***	0.30±0.30	0.82±0.82	1.67±1.67***
**MCP-1**	12.95±1.12	88.56±15.07	945.24±78.74***	12.03±0.50	181.47±57.40	516.03±79.26***[bbb]
**MIP-1α**	15.89±3.32	51.66±8.30	267.64±24.6***	11.04±1.65	79.87±22.59	210.60±21.83***
**MIP-1β**	51.00±5.35	146.87±24.30	527.84±38.51***	38.54±5.06	220.65±52.16*	410.06±46.02***
**MIP-2**	6.38±0.64	11.10±1.00	29.40±1.25***	5.28±0.30	12.97±2.46*	23.43±1.26***[b]
**MIP-3α**	11.70±0.83	19.82±3.32	68.88±9.32***	10.32±1.39	19.17±3.69	40.55±7.70*[bb]
**Eotaxin**	106558.40±7063.18	99877.50±7408.43	135373.40±8793.24	95990.00±3951.17	96833.00±9621.77	106184.30±6467.64[b]
**RANTES**	1263.75±240.27	708.30±61.31*	860.52±188.97	588.86±131.77*	567.38±117.95**	544.85±44.79**[bb]
**SDF-1α**	23.50±3.53	53.47±7.98	124.38±17.43***	21.60±7.74	65.30±16.72	85.86±13.16*
**TARC**	137.58±12.69	127.65±10.17	118.79±16.79	109.56±0.74	91.87±15.97	97.00±12.27
**6CKine**	305.27±7.56	330.99±10.10	1010.71±103.32	353.27±27.47	371.06±10.19	692.35±89.56[bb]
**TH2/Regulatory Cytokines**						
**IL-21**	949.29±46.74	1,102.24±120.14	1,455.73±189.17	712.38±44.53	1,102.51±118.68	1,432.13±136.16
**IL-22**	5.57±0.40	9.15±1.46	20.56±2.49***	4.88±0.22	10.54±1.91	15.54±1.08**
**IL-9**	10.70±0.88	28.89±3.18	116.88±6.24***	8.05±0.81	34.49±7.96	77.46±6.53***[bbb]
**IL-10**	1.70±1.05	3.34±0.50	4.51±3.67	3.05±0.29	3.71±0.98	4.60±1.02
**IL-13**	0.01±0.01	0.08±0.05	0.50±0.40*	0.01±0.01	0.04±0.02	0.67±0.13**
**IL-4**	26.08±26.08	53.57±10.59	166.80±133.54	7.86±6.50	121.67±14.69[aa]	215.03±55.55**
**IL-15**	121.80±74.59	703.03±88.63	1,975.27±746.58***	409.78±161.26	886.37±157.06	1,782.20±506.48***

**IL-17F**	570.04±81.63	388.43±52.76	517.83±333.04	299.63±61.04	434.54±97.83	843.75±64.90
**IL-23**	2.82±0.39	2.06±0.33	2.47±1.24	1.26±0.12	2.23±0.26	3.06±0.34
**IL-27p28**	270.95±201.06	482.49±138.53	1,385.71±874.80	647.92±263.76	1,259.99±251.67[a]	1,303.05±640.17
**IL-31**	463.202±211.85	339.23±133.99	3,063.87±955.29***	467.372±209.84	1,873.42±292.31*[aa]	1,967.68±604.11*
**IL-33**	252.46±112.86	300.80±50.10	699.11±444.92	255.12±73.59	200.89±58.97	962.79±186.08**
**Growth Factors/Hormones**						
**GM-CSF**	1.06±0.07	1.44±0.08	4.10±0.20***	1.01±0.05	1.43±0.18	2.87±0.19***[bbb]
**VEGF**	79.28±44.64	101.27±30.56	316.64±94.83**	39.40±13.42	119.74±22.89	280.07±11.30**
**Erythropoietin**	B.D	0.02±0.02	1.43±0.49**	B.D	B.D	0.36±0.24[b]
**BAFF**	7718.83±133.49	9457.37±351.86	11503.07±299.39	9998.84±261.39[a]	9899.54±522.93	11304.99±1059.52

Data are shown as mean concentration (pg/mL) ± SEM (n=5-10 mice per group pooled from 2 experiments) for all cytokines except BAFF, NGAL, and 6CKine (n=4 per group). B.D. indicates below the detection limit of the assay. *p<0.05, **p<0.01, ***p<0.001 indicates significance change in cytokine/chemokine level over time relative to uninfected and vehicle treated control. [a]p<0.05, [aa]p<0.01, [aaa]p<0.001 indicates significance between vehicle and FPS treatment in SARS-CoV-2 infected mice on day 2 post infection. [b]p<0.05, [bb]p<0.01, [bbb]p<0.001 indicates significance between vehicle and FPS treatment of SARS-CoV-2 infected mice on Day 4 post infection. Comparisons between groups were assessed using a 2-way ANOVA with Dunnett's post test.
